# Procalcitonin as a marker of sepsis and outcome in patients with neurotrauma: an observation study

**DOI:** 10.1186/1471-2253-13-48

**Published:** 2013-12-15

**Authors:** Shuixiang Deng, Hechen Zhu, Kunlun Wang, Tongwa Cao

**Affiliations:** 1 Division of Intensive Care Unit, Huashan Hospital, Fudan University, 12 Wulumuqi Road (middle), Shanghai, China; 2 Department of emergency, Jinshan Hospital, Fudan University, Longhang Road, Shanghai, China

**Keywords:** Procalcitonin, Systemic inflammatory response syndrome (SIRS), Sepsis, Mortality, Traumatic brain injury

## Abstract

**Background:**

Procalcitonin (PCT) is a reliable biomarker of sepsis and infection. The level of PCT associated with sepsis and infection in patients with traumatic brain injury is currently unknown. The purpose of this study was to investigate the value of PCT and C-reactive protein (CRP) as diagnostic markers of sepsis and to evaluate the prognostic value of these markers related to the severity of injury, sepsis and mortality.

**Methods:**

105 adult patients with neurotrauma were enrolled in this study from June 2011 to February 2013. PCT and CRP were measured at admission and 2, 3, 5 and 7 days after admission. The sepsis criteria established by American College of Chest Physicians /Society of Critical Care Medicine Consensus Conference were used to identify patients. Injury Severity Score (ISS) and Glasgow Coma Score (GCS) were used to assess the severity of the injury. All these patients were monitored for 28 days.

**Results:**

At admission, the median level of PCT was consistent with the severity of brain injury as follows: mild 0.08 ng/ml (0.05 - 0.13), moderate 0.25 ng/ml (0.11 - 0.55) and severe 0.31 ng/ml (0.17 - 0.79), but the range of CRP levels varied greatly within the given severity of brain injury. Seventy-one (67.6%) patients developed sepsis. The initial levels of PCT at admission were statistically higher in patients with sepsis, compared with patients with systemic inflammatory response syndrome (SIRS), but there were no differences in the initial concentration of CRP between sepsis and SIRS. After adjusting for these parameters, multivariate logistic regression analysis revealed that PCT was an independent risk factor for septic complications (p < 0.05). The areas under the ROCs at admission for the prediction of mortality were 0.76 (p < 0.05) and 0.733 for PCT and CRP, respectively.

**Conclusions:**

Increased levels of PCT during the course of the ICU stay could be an important indicator for the early diagnosis of sepsis after neurotrauma. In addition, high serum levels of PCT in patients with neurotrauma at admission indicate an increased risk of septic complications, and the daily measurement of PCT assists in guiding antibiotic therapy in neurotrauma patients.

## Background

Traumatic brain injury (TBI) accounts for a large proportion of injury-related deaths and disabilities in developed countries [1]. Patients with TBI have an increased risk of consequent infection and sepsis, which requires prompt diagnosis and treatment with appropriate antimicrobial agents to reduce associated morbidity and mortality [2-4]. Severe neurotrauma is a great potential cause of systemic inflammatory response syndrome (SIRS) [5], and the currently available markers including fever, C-reactive protein (CRP), IL-6 and total leukocyte count lack sensitivity and specificity thereby making it difficult to distinguish SIRS from infectious diseases [6]. Reducing the risk of infection and subsequent sepsis through adherence to infection control measures is essential to lower in-hospital deaths among patients with TBI.

In the last decade, several papers have been published that report procalcitonin (PCT) as a novel biomarker that is recommended for the assessment of bacterial infection [7-11]. PCT is composed of 116 amino acids and is physiologically synthesised by thyroid C cells, but in the case of sepsis, there is an extrathyroidal origin of PCT genesis [7,8]. In normal conditions, serum PCT levels are negligible, but they become detectable at the onset of infection. PCT levels are closely related to the severity and evolution of infection, and they are thought to be associated with poor prognosis in patients with septicaemia [12,13]. PCT has been used to evaluate the evolution of infections and sepsis in patients with trauma and surgical conditions [5,14,15]. The changes of PCT level in response to therapeutic treatment have also been reported, which suggests prognostic significance in a variety of clinical settings [16,17]. The persistent increase of PCT level is associated with an increased length of ICU stay and mortality [18]. PCT-guided strategies have significantly reduced the use of antibiotics [19]. Among the proposed biomarkers of sepsis, PCT appears to be the most promising in terms of its diagnostic and prognostic benefits. Recently, a newly developed PCT assay with significantly higher discriminatory power has been put into practice [20]. There is a lack of substantial data regarding the use of PCT in the treatment of traumatic brain injury [5,21].

The aim of this study was to investigate the levels of PCT and CRP that are used as diagnostic markers of sepsis in patients with traumatic brain injury at the time of hospital admission, and to evaluate the prognostic value of these markers related to the severity of injury, sepsis and mortality.

## Methods

### Patients and methods

After approval by the Ethics Committee of Huashan Hospital, Fudan University, Shanghai, China (approval number: 2011–281), informed consent was obtained from each patient or their representatives. A total of 105 patients with isolated traumatic brain injury who were admitted to the ICU of our tertiary university teaching hospital from June 2011 to February 2013 were enrolled in this prospective observational study. Patients were included if they fulfilled the following criteria: age over 18 years old and admission within 24 hours of injury. Patients with pre-existing febrile illness, suffering from burns, an Abbreviated Injury Scale (AIS) for all other body regions injury ≥ 3, patients under immune suppressive therapy, patients already on antibiotics for ≥3 days before admission, and patients who did not survive for 48 hours after admission were excluded from the study.

### Clinical data collection

All patients in the ICU were monitored by ECG and arterial pressure monitoring as a part of routine clinic practice. The clinical care of the patients was guided by the criteria established by the Brain Trauma Foundation and the American Association of Neurological Surgeons [22-24].

Endotracheal intubation was carried out, and mechanical ventilation was initiated as clinically required. Glasgow Coma Score (GCS) and Injury Severity Score (ISS) were used to define the injury severity [25,26]. GCS and ISS were calculated within the first 24 hours after admission and were repeated every day thereafter. The levels of PCT and CRP were measured on days 1 (admission day), 2, 3, 5 and 7. All data including clinical signs, laboratory observations, microbiological pathogens, medicine options, complications, 28-day survival rate and duration treatment and length of stay in ICU as well as the data necessary to evaluate the ISS were documented.

### Definitions

The main complication was systemic inflammation, and the various stages of sepsis were defined according to the criteria established by the American College of Chest Physicians/the Society of Critical Care Medicine [6]. We evaluated the onset of sepsis during the first observation week. Patients were allocated to four groups post hoc: (1) NoSIRS (neither SIRS nor sepsis) (2) SIRS (3) sepsis and (4) severe sepsis group (including severe sepsis and septic shock).The stratification of trauma severity in terms of the GCS score was generally recognised and accepted by professionals [25]. Then, patients were allocated to three groups post hoc: (1) GCS13-15 group (2) GCS9-12 group and (3) GCS3-8 group. The patients who survived longer than 28 days were considered as survivors.

### Measurement of plasma PCT and CRP

According to the manufacturer’s instructions, PCT level was measured by an electrochemiluminescence immunoassay (ECLIA) B.R.A.H.M.S. PCT ELECSYS® using an automated Roche Elecsys and Cobase immunoassay analyser [20]. This new assay is more sensitive compared with the conventional assays, and the sensitivity of this assay is 0.02 ng/ml. CRP level was determined using a fully automated IMMAGE Immunochemistry System (Beckman Coulter, USA), which was derived from the highly sensitive near infrared particle immunoassay method. The lower limit of detection is 3.45 mg/L.

### Statistical analysis

Normally distributed variables are presented as the mean +/− standard deviation (SD), and nonparametric continuous variables are expressed as the median and inter-quartile ranges (IQR). PCT levels among sepsis groups or GCS groups were carried out using Kruskal-Wallis tests (data not normally distributed), if statistical significance of differences was detected, then the Mann–Whitney U-test (nonparametric analysis) was used for further comparisons between the two sepsis groups or GCS groups. We used the Pearson chi-square test (χ^2^ test) or Fisher’s exact test to compare proportions. Multivariate logistic regression was used to assess the performance of the variables in the prediction of sepsis. Based on the results of univariate analysis, we selected three confounding variables (age (p = 0.789), sex (p = 0.779), ISS (p < 0.05)) that required adjustment to minimise their influence on the results. Receiver operating characteristic curves (ROC) and the area under the curve (AUC) were employed to assess the predictive performance of the models. A two-tailed p value < 0.05 was considered to be statistically significant. Statistical evaluation of data was analysed using the SPSS version 17.0 software.

## Results

### Demographics and clinical characteristics of patients

One hundred five isolated traumatic brain injury patients without underlying illness or long-term medication were admitted within 24 hours after accident. Among these patients, 90 (85.7%) were traumatised by motor vehicle, whereas 15 casualties were suffered from trauma following a fall from a greater height. The mean age of the patients was 56 years and ranged from 18 to 80 years with 79 males and 26 females. The demographics and clinical characteristics of patients are presented in Table 1.

**Table 1:** Demographic data of series of patients by GCS subgroup

**Parameters**	**GCS13**-**15Group**	**GCS9**-**12Group**	**GCS3**-**8Group**	**Totality**	***p value***
Number (n)	33	38	34	105	-
Age (mean ± SD)	55 ± 14	56 ± 14	55 ± 14	55 ± 13	1.0
Gender (male/female)	21/12	31/7	27/7	79/26	0.172
ICU stay(d)	12.4 ± 5.5	15.2 ± 9.6	16 ± 7.1	14.6 ± 7.7	0.125
Initial ISS	4 ± 3	13 ± 5	17 ± 5	12 ± 7	0.001
Initial C-reactive protein (0–24 hours) (median, quartiles) (mg/l)	10.23(3.5,15.9)	23.75(13.3,42.5)	44.85(22.4,78.1)	23.1(10.3,46.1)	0.244
Initial procalcitonin (0–24 hours)(median, quartiles) (ng/ml)	0.08(0.05,0.13)	0.25(0.11,0.55)	0.31 (0.17,0.79)	0.2(0.08,0.5)	0.110
Mortality (%)	1.0	2.9	11.3	15.2	0.000

### Generation of PCT and CRP after trauma

**Figure 1:**
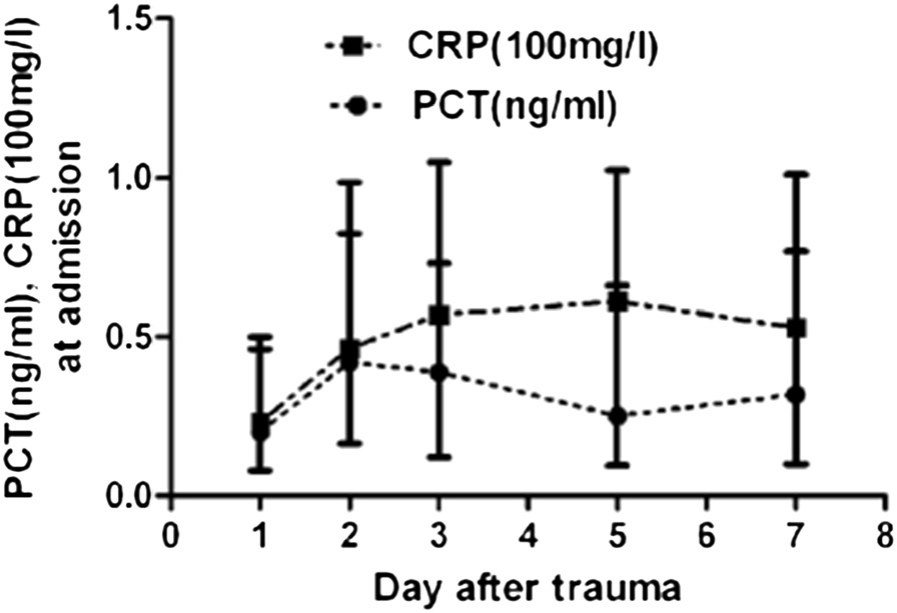
**Serum levels of procalcitonin (PCT) and C-reactive protein (CRP) in patients with neurotrauma.** Time course of induction (median, quartiles).

The median levels of serum PCT and CRP in the overall patient cohort were 0.2 ng/ml and 23.1 mg/L, respectively. After comparisons were carried out among the different groups that were divided according to their GCS scores, an incremental increase in the median level of PCT that was consistent with increasing severity of brain injury was observed. The median level of PCT in patients with mild brain injury was significantly lower than that in patients with moderate and severe brain injury. The dynamic changes of PCT and CRP levels after neurotrauma are depicted in Figure 1.

### Development of sepsis

**Figure 2:**
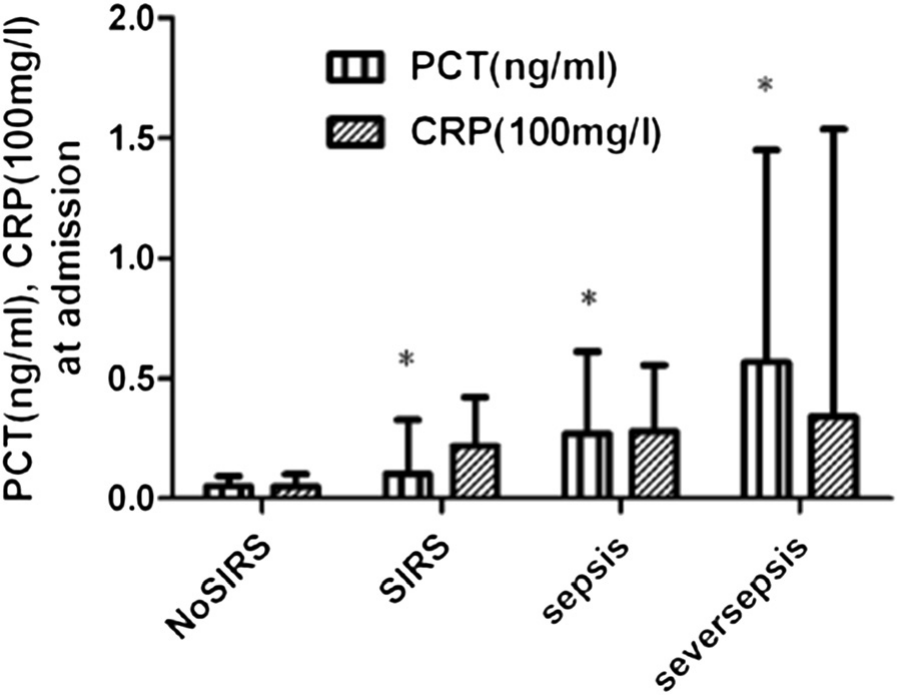
**The initial serum concentration of procalcitonin (PCT) and C-reactive protein (CRP) after neurotrauma and the development of septic complication during a 28-day follow-up.** SIRS: systemic inflammatory response syndrome, *p < 0.05, Mann–Whitney U test.

**Figure 3:**
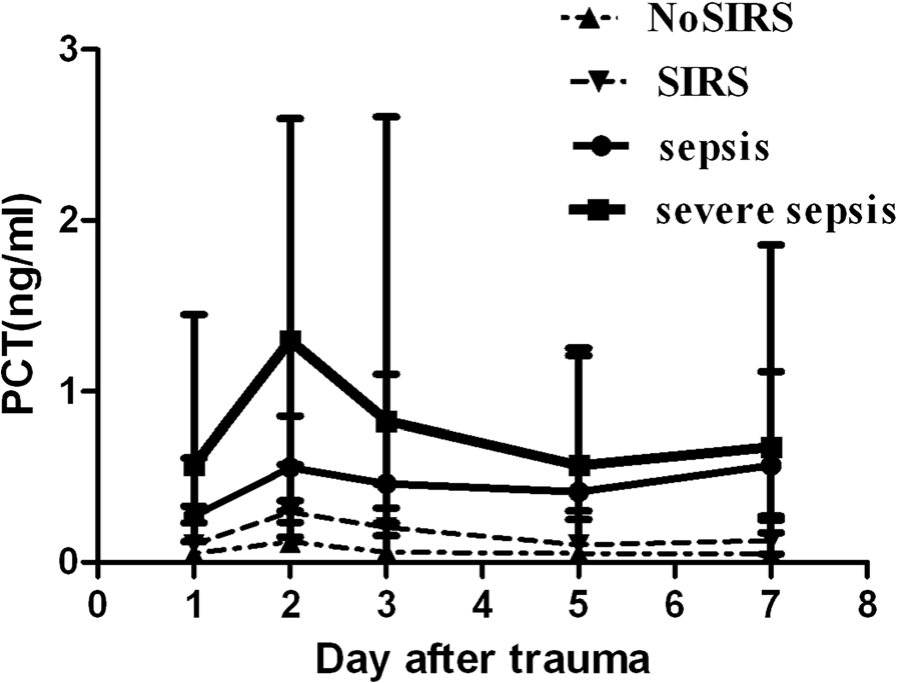
**Course of procalcitonin (PCT) in patients who developed various stages of severe sepsis, sepsis, systemic inflammatory response syndrome (SIRS), or NoSIRS (median**, **quartiles).**

The incidence of SIRS or sepsis occurred in 79% patients with traumatic brain injury. Among 105 patients, 71(67.6%) were clinically diagnosed as septic including 15 patients (14.3%) who suffered from severe sepsis or septic shock. Positive blood culture was detected in 43 pneumonia patients, 10 peritonitis patients, 9 urinary tract infection patients, 7 wound infection patients and 8 patients with infection of other parts of body. Sepsis, severe sepsis or septic shock was frequently and directly diagnosed during the observation period in patients with initially high levels of serum PCT. On the contrary, the initial level of CRP was not associated with these categories of infection as shown in Figure 2 and Table 2. For example, the patients PCT level with 0.05 ng/ml that ranged from <0.03 to 0.098 ng/ml initial median (quartiles) did not develop SIRS or sepsis during the entire course of observation. Conversely, the patients with 0.105 ng/ml that ranged from 0.085 to 0.328 ng/ml initial median PCT level developed SIRS (p = 0.002), and those with 0.27 ng/ml that ranged from 0.12 to 0.61 ng/ml initial median PCT level developed sepsis (p = 0.001). The patients with 0.57 ng/ml that ranged from 0.23 to 1.45 ng/ml initial median PCT level developed septic shock (p < 0.001). There was a significant difference in the initial level of PCT between SIRS and sepsis (p = 0.046). PCT concentration remained elevated in patients with sepsis, severe sepsis or septic shock, but it rapidly fell to a near-normal value in the patients who did not develop sepsis (Figure 3). There was a significant difference in the median level of PCT between the measurements at admission and 7 days after admission (0.32 vs 0.2 ng/ml, p < 0.002). Univariate logistic regression analysis indicated that the initial white blood cell count (WBC), CRP and PCT were the high risk factors for sepsis/severe sepsis or septic shock, and after adjusting for these parameters, multivariate logistic regression analysis indicated that the odds ratio for the development of sepsis/severe sepsis or septic shock increased if PCT was >0.215 ng/ml, but other markers had no statistical significance (Table 3).

**Table 2:** The original data of PCT and CRP in patients of sepsis subgroup

**Group**	**PCT (ng/ml)(d1)**		**CRP (mg/L)(d1)**		**PCT (ng/ml)(d2)**	
	**Median**	**IQR**	**Median**	**IQR**	**Median**	**IQR**
No SIRS	0.093	(0.050,0.093)	4.86	(3.22,10.30)	0.120	(0.080,0.300)
SIRS	0.105	(0.085,0.328)	21.90	(14.48,42.22)	0.295	(0.148,0.575)
Sepsis	0.270	(0.120,0.610)	27.85	(15.12,55.50)	0.555	(0.233,0.857)
Severe sepsis	0.570	(0.230,1.450)	34.30	(22.40,154.0)	1.300	(0.360,2.600)

**Table 3:** Multivariate logistic regression analysis of the risk factors of sepsis

	**B**	**S.E**.	**Wald**	**p**	**odds ratio**
Initial WBC	0.062	0.077	0.661	0.416	1.064
Sex	0.580	0.836	0.481	0.488	1.787
Age	0.010	0.027	0.148	0.700	1.010
initial ISS	0.352	0.084	17.694	0.000	1.422
Initial CRP	0.016	0.013	1.600	0.206	0.984
Initial PCT	5.672	2.399	5.589	0.018	290.681

### Serum PCT and prognosis

Of the 105 patients, 16 died of severe head trauma with a GCS score of 3–8 within 28 days after trauma, which accounted for 15.24% mortality. The initial PCT and CRP levels after trauma were significantly higher in nonsurvivors than those in survivors (p < 0.05 by Mann–Whitney U-test). However, serum PCT was superior compared to CRP in the prediction of mortality with a greater area under the ROC curve (0.76 vs 0.733).

## Discussion

TBI patients present a particular challenge in the diagnosis of sepsis complications as the trauma per se predisposes patients to provoking a systemic inflammatory response that often masks the initial clinical symptoms of sepsis. Therefore, patients with TBI are considered to be at a high risk for sepsis complications, which complicate the ability to distinguish sepsis from SIRS in a clinical setting by using ordinary clinical signs and symptoms such as WBC, high fever and mal-perspiration. Alternatively, PCT is considered as an acute-phase biomarker of the systemic inflammatory response [27]. We carried out this study to determine whether PCT and CRP can be diagnostic markers of sepsis or prognostic indicators of mortality of neurotrauma patients.

In the present study, PCT level increased in the first 24 hours after trauma in patients with GCS < 12. However, the median PCT level in patients with GCS 9–12 was lower than that of the patients with GCS < 8. Various levels of PCT and CRP were detected in patients with TBI. Both biomarkers showed that the variation of CRP levels at different intervals after trauma was a uniform response without significant association with trauma severity. The development of various stages of sepsis was also observed. Furthermore, the concentration of CRP remained elevated for several days after trauma. The variation of PCT levels was moderately consistent with the severity of the trauma that was previously reported by several authors [5,28,29]. Of particular note, the initial median level of PCT was closely associated with the severity of TBI. The mechanism of this phenomenon could be explained by the observation that SIRS is a more common occurrence that is promptly activated in neurotrauma patients or that the PCT level is assayed by a sophisticated instrument, therefore, this phenomenon diminished the value of PCT being used as a specific marker of sepsis in these patients.

As noted, the median PCT, but not CRP, level was determined to be significantly higher in patients with sepsis, severe sepsis or septic shock compared to the levels in patients with SIRS after the evaluation of patients according to criteria established by the ACCM/SCCM. CRP is another acute inflammatory protein and it often takes a long time to activate a reaction whereby its levels are elevated in response to inflammation. Studies have shown that the kinetics of CRP in multiple-trauma is slower and sustains longer than PCT [30]. These findings could be due mainly to the low sensitivity of CRP that is responsive to trauma at an early stage. According to the multivariate logistic regression analysis, after adjustment for age and gender, the PCT value was an independent risk factor of sepsis. Patients with high initial PCT levels were subjected to a 290-fold risk of sepsis compared to those with a low initial PCT level.

There are several studies that have reported using PCT to guide antibiotic therapy in different settings [31-33]. Experts have reached a consensus and developed guidelines for the clinical interpretation of elevated PCT and the risk stratification according to different elevated PCT levels. In particular, the negative predictive value (PCT < 0.1 ng/ml) to exclude a risk of sepsis is used. In the present study, we conventionally administered a single shot antibiotic to neurotrauma patients on admission to prevent infection. We determined that the odds ratio for the development of sepsis was increased as PCT > 0.215 ng/ml, and according to the daily measurement of the PCT value, if PCT remains lower than 0.1 ng/ml, further antibiotic treatment was not required due to the low risk for sepsis. This finding was consistent with Marc and colleagues [33]. We also determined that PCT concentration rapidly decreased to a near-normal value in patients who did not develop sepsis, thus, during the further course of treatment, if the PCT level remained <0.1 ng/ml and the combined clinical symptoms did not provided any evidence for sepsis, no antibiotics were required. Thus, the daily use of the negative predictive value of PCT would be clinically helpful.

Our study has several important implications for clinicians. Although the present study population is too small to infer the importance of serum PCT in neurotrauma patients, it definitively indicates that serum PCT could be involved in the entire course of infections to facilitate the management of sepsis in critical care. With the newest assay method, serum PCT is detected with a high accuracy that other currently available tests cannot provide. The accuracy of the serum PCT reference range is not perfect, but it could guide physicians in developing a clinical strategy and incrementally managing neurotrauma patients with sepsis. Additionally, the daily measurement of PCT aids physicians in guiding antibiotic therapy in neurotrauma patients. The test can be performed within 30 minutes and provides valuable information long before culture results are available.

There are some limitations to the present study. First, the sample size of the study was relatively small, and consequently, the power to demonstrate the interaction among serum PCT and prognosis was limited. Second, most patients were young males, which did not represent the entire demographics of neurotrauma patients. Third, the present study did not analyse the kinetics of PCT level and its association with other inflammatory cytokines levels in these patients. Finally, the pathophysiology of neurotrauma was complex and was influenced by patient-specific factors (age, sex) and injury-specific factors (mechanism, severity). Thus, one single biomarker will not be able to accurately predict the clinical course of neurotrauma patients.

## Conclusions

These data substantiate the hypothesis that increased levels of PCT during the period of hospitalisation could be an important indicator for the early recognition of bacterial infection and sepsis as well as provide early information about the presence of complications after TBI. These data also indicate that the daily measurement of PCT would be clinically useful. The authenticity and soundness of the findings obtained from our study need to be evaluated in a future study.

## Competing interests

The authors declare that they have no competing interest.

## Authors’ contributions

TC developed the study design and coordinated its implementation. SD participated in data collection and interpretation/discussion of results and drafted and revised the manuscript. HZ participated in guiding the study design and revised the manuscript. KW was responsible for patient recruitment as well as data collection and carried out the statistical analysis. All authors read and approved the final manuscript.

## Pre-publication history

The pre-publication history for this paper can be accessed here:

http://www.biomedcentral.com/1471-2253/13/48/prepub
